# Influence of Thermal Annealing Temperatures on Powder Mould Effectiveness to Avoid Deformations in ABS and PLA 3D-Printed Parts

**DOI:** 10.3390/polym14132607

**Published:** 2022-06-27

**Authors:** Joaquín Lluch-Cerezo, María Desamparados Meseguer, Juan Antonio García-Manrique, Rut Benavente

**Affiliations:** 1 Department of Mechanical Engineering and Materials, Universitat Politècnica de València, Camino de Vera s/n, 46022 Valencia, Spain; amesegue@mcm.upv.es (M.D.M.); jugarcia@mcm.upv.es (J.A.G.-M.); rutbmr@upvnet.upv.es (R.B.); 2 Engineering Research Team, Florida Universitària, 46470 Catarroja, Spain; 3 Instituto de Tecnología de Materiales, Universitat Politècnica de València, Camino de Vera s/n, 46022 Valencia, Spain

**Keywords:** fused deposition modelling (FDM), annealing, powder mould, flexural strength, acrylonitrile butadiene styrene (ABS), polylactic acid (PLA)

## Abstract

Fused deposition modelling (FDM)-printed parts can be treated with various post-processes to improve their mechanical properties, dimensional accuracy and surface finish. Samples of polylactic acid (PLA) and acrylonitrile butadiene styrene (ABS) parts are treated with annealing to study a ceramic powder mould’s effectiveness in order to avoid dimensional part deformation. The variables chosen are annealing temperatures and the usage of a ceramic powder mould to avoid part deformations. A flexural strength test was carried out to evaluate the mould’s influence on the mechanical properties of the part. The effectiveness of the mould has been evaluated mainly attending to the length of the part, because this is the dimension most affected by deformation. A polynomial approximation to a deformation’s length and the effectiveness of the mould allows for their prediction. Results obtained show that effectiveness increases with the annealing temperature. Nevertheless, mould effectiveness decreases when parts are fabricated with PLA, because it is a semi-crystalline thermoplastic, and it suffers a lower shrinkage during thermal post-process than amorphous polymers such as ABS. Attending to the flexural strength test, mould has no significant influence on the mechanical properties of the treated parts in both materials studied.

## 1. Introduction

Additive manufacturing (AM) is becoming a technological revolution into industrial processes. These technologies have great potential, and their number of industrial applications is widely increasing [1]. One of the most widely used and affordable AM techniques is fused deposition modelling (FDM) [2]. This technology has several advantages, such as the capability to produce complex shapes without manufacturing restraints, design flexibility, low equipment and material costs and a wide range of plastic materials, as well as the ability to manufacture large-scale components [3]. As a result, FDM has emerged as a flexible and powerful technique in the advanced manufacturing industry. This technology is becoming widely used as a manufacturing process in both aerospace and automotive industries [4]. However, 3D printing processes present numerous challenges in its applications and clarify the advantages and disadvantages, and comparison with conventional manufacturing methods is needed [5].

FDM technology allows for the use of a wide range of thermoplastic types. Acrylonitrile–butadiene–styrene (ABS) and polylactic acid (PLA) are among the most widely used thermoplastics due to their availability and easy machinability [6]. These materials are representative of two major groups of thermoplastics, amorphous and semi-crystalline, and they are commonly used for experimentation by FDM process researchers. PLA is a semi-crystalline thermoplastic used in a wide range of applications, from biodegradable packing and disposables to medical implants and personal care products. ABS is an amorphous polymer widely used in industrial applications, from automotive to pipes and fittings. It is comparatively more challenging to print than PLA. Nevertheless, ABS is also more suitable for UV radiation or high-temperature applications compared to PLA.

Scientific literature clearly shows that mechanical properties of FDM parts are largely influenced by variable process parameters [6,7,8,9,10,11,12,13,14], such as printing speed, printing and build plate temperatures, density and pattern infill lines, layer height and layer width. These variables can be optimised to obtain the desired mechanical response, dimensional accuracy and part quality [15,16,17]. However, in some industrial FDM applications, typical anisotropy of the FDM process and low dimensional accuracy can inhibit its use as functional parts, and its applications remain a significant challenge [18]. In addition, other problems must be considered. In the FDM process, plastic material is heated quickly, extruded, and then cooled rapidly in contact with material extruded before in other layers. Due to the poor heat conduction of the plastic, rapid heating and cooling create internal stress in the printed part and result in the shrinkage of the layers. Temperature profile theoretical modelling can help to clarify heat transfer between layers and can be valuable in more accurately predicting the internal stress and adhesion of layers [19]. Additionally, in FDM techniques, there is a high chance of weak layer-to-layer adhesion as well as the formation of voids between the fabricated layers, which leads to reduced part quality [20]. In order to achieve the desired mechanical properties of the part, in some cases, in addition to optimizing the process parameters, it is necessary to apply post-processing techniques to improve them.

FDM-printed parts can be treated with various post-process to improve mechanical properties, dimensional accuracy and surface finish. These post-processes can be classified as mechanical, such as machining, polishing and sanding, and as chemical, such as annealing, remelting, steam smoothing, gap filling and epoxy coating [21,22,23]. One of the most promising post-processing techniques is annealing. This post-process enhances the tensile strength and strain by increasing the percentage of crystallinity, reducing air gaps, improving layer-to-layer adhesion and removing internal stress [24,25,26,27]. Annealing post-process effects differ between amorphous and semi-crystalline polymers. ABS parts mainly improve quality characteristics due to a material reflow, causing interlayer gaps to be filled as well as better inter-layer bonding [28,29]. On the other hand, PLA parts mainly increase flexural stress due to increasing crystallinity. This effect depends on annealing temperature and heating and cooling times [25,30].

However, thermal post-processing could affect the dimensional tolerances of FDM-printed parts and even lead to unacceptable deformations that could affect the usability of the part [31]. In order to avoid deformations during the thermal post-process, some studies have packed FDM parts in salt powder [32,33] or ceramic alumina powder [34]. Powder mould has been used in annealing and remelting post-process. In remelting, the powder adheres to the surface of the part. In this case, salt is much easier to remove than ceramic powder. In contrast, in an annealing process, the melting temperature is not reached, the powder does not adhere, and it is more advisable to use an inert powder such as alumina. The effectiveness of using a ceramic powder mould to avoid deformations of PLA parts in 135 °C annealing post-process was investigated by Lluch et al. [34], who concluded that using a ceramic powder mould considerably reduces dimensional deformations in the post-process. Due to the results obtained in this study, the research will be extended to other materials with a wide range of annealing temperatures.

In this study, ABS and PLA thermoplastics are selected as manufacturing materials. Dimensional part deformation in post-process thermal treatment is studied, in addition to the influence of annealing temperature on the effectiveness of the mould to avoid part deformations. A polynomial approximation of the results has been used to predict part deformations during the annealing post-process and mould effectiveness at a wide range of temperatures. To reach this objective, a variation of part dimensions (length, width and height) will be measured according to annealing temperature. Mechanical property flexural strength of ABS and PLA are studied, as well as how they can be influenced by the use of the mould during the annealing process.

## 2. Materials and Methods

### 2.1. Test Specimens Design and Manufacturing

Samples are designed according to standard ISO 178:2019 [35] in order to determine the flexural properties. This geometry allows for an easy analysis of the dimensional variations occurring during the annealing post-process.

**Figure 1:**
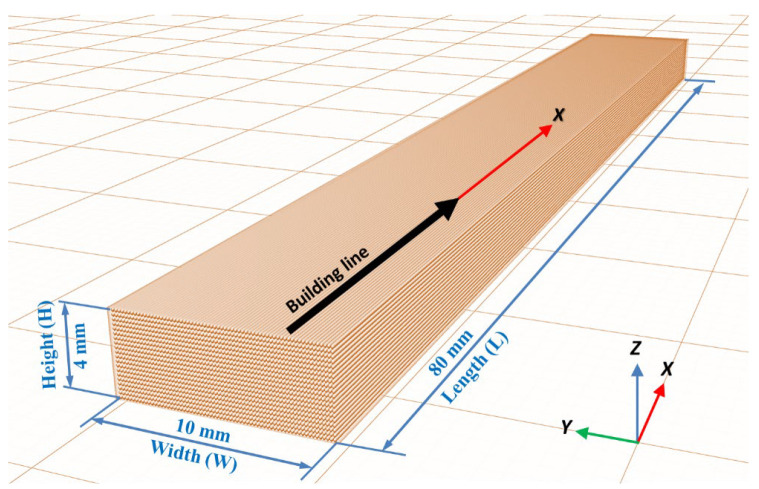
Specimen dimensions and building line direction.

ABS and PLA samples are manufactured using a FDM 3D printer Ultimaker 3 Extended (Ultimaker B.V., Utrecht, The Netherlands) equipped with a 0.4 mm-diameter nozzle. All building lines of each specimen are printed without differences between infill lines, wall lines and bottom and top layer lines. Unidirectional building lines printed in 0 degrees orientation in the XY plane (code XY + 0 according to [36]) with a 100% density infill line pattern are chosen for all samples. Specimen dimensions are 80 mm × 10 mm × 4 mm. (Figure 1).

Three-dimensional-printed specimen geometry is modelled using Inventor software (Autodesk, Inc., San Rafael, CA, USA). Cura software (Ultimaker B.V., Utrecht, The Netherlands) was used to generate G-code files and to command and control all the FDM 3D printing parameters.

In order to reduce the interaction between lines of the same layer, a layer height of 0.2 mm and a line width of 0.5 mm values are selected. In the 3D manufacturing process, printing parameter values are shown in Table 1.

The commercial 2.85 mm White ABS and Pearl White PLA 3D Printer Filaments Materials (Ultimaker B.V., The Netherlands) are used. The values of the main thermal properties of ABS and PLA material used in this paper are the following:

- The ABS melting temperature range is 225–245 °C (test method according to ISO 294-1:2017 [37]), and the Vicat softening temperature is 97 °C (test method according to ISO 306:2013 [38]).
- The PLA melting temperature range is 145–160 °C (test method according to ISO 11357-3:2018 [39]), and the glass transition temperature is 60 °C (test method according to ISO 11357-2:2013 [40]).

### 2.2. Thermal Post-Process Treatment

**Figure 2:**
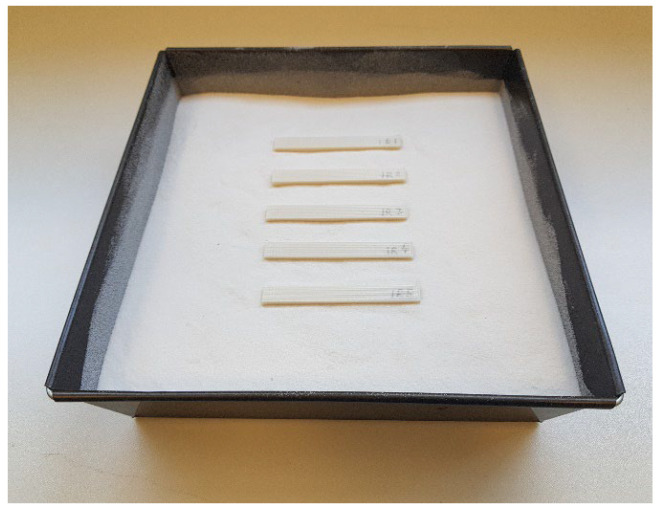
Sample of 5 specimens placed inside the mould.

The thermal post-process described by Lluch et al. [34] has been followed. The samples to be treated are introduced inside the mould over a 1 cm layer of dry alumina powder with an average grain size of 150 μm, (Protechno, Spain) (Figure 2) and covered by another powder layer of the same thickness. A pressure of 12 g/cm^2^ to avoid creep deformations is exerted on the alumina powder. No binders are used, as the pressure is sufficient for the ceramic powder to behave as a solid.

Samples are heat-treated in a convection furnace at different temperatures above the ABS Vicat softening temperature and the PLA glass transition temperature, respectively, for 120 min. A 10 °C/min ramp is used to ensure that the temperature inside the mould is as uniform as possible. Before unpacking samples, the mould was kept in the furnace until it reached room temperature.

### 2.3. Design of Experiments

The variables used to study the dimensional deformations and flexural strength after the thermal treatment are the material (ABS or PLA), the annealing temperature and the usage of a ceramic powder mould. The material and the usage of ceramic mould are studied at two levels, and the annealing temperature is studied at seven levels (Table 2).

Annealing temperatures are different for the studied materials. The temperature range starts at 3 °C above Vicat for the ABS material and at the glass transition point for PLA, and it ends near the temperature fusion point for both materials. Table 2 shows the annealing temperatures for ABS (without brackets) and PLA (in brackets).

A full factorial design is used to determine the combination of variable levels to use for each experimental case (Table 3). Five replicas of each experiment are performed. A total of 140 specimens (70 ABS and 70 PLA) were printed at 0 degrees in the XY plane orientation, according to Figure 1.

### 2.4. Dimensional Analysis

**Figure 3:**
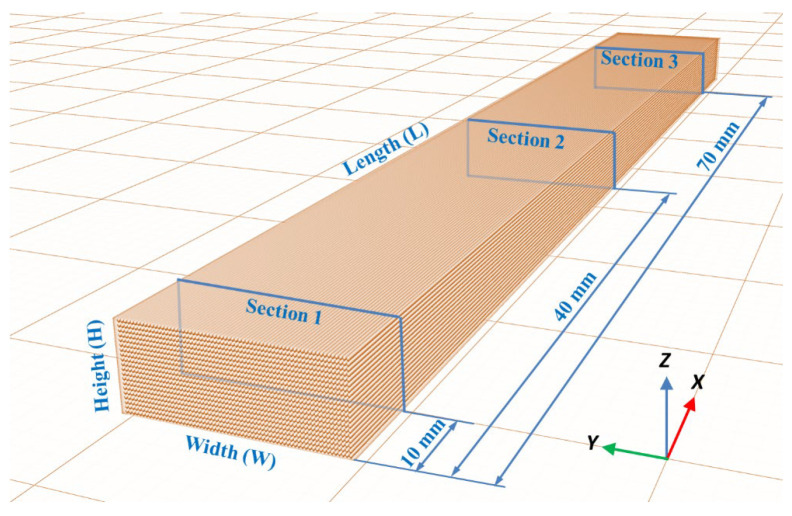
Measured sections in each specimen.

Samples’ deformation due to thermal post-processing is determined in all three dimensions of the material. The length (L), width (W) and height (H) values of each specimen before and after heat treatment are measured to evaluate dimensional changes. The height (H) and width (W) values are obtained as an average of the values in three sections of each specimen (Figure 3). Measurements are carried out according to standards using an electronic digital caliper instrument (resolution = 0.01 mm, accuracy = ±0.03 mm).

*L*, *W* and *H* variation are calculated according to ISO 294-4:2018 [41] using Equations (1)–(3). The subscripts “*f*” and “*o*” indicate “after” and “before” thermal post-processing, respectively.

(1) $$\Delta L=100\cdot \frac{L_{f}-L_{o}}{L_{o}}$$

(2) $$\Delta W=100\cdot \frac{W_{f}-W_{o}}{W_{o}}$$

(3) $$\Delta H=100\cdot \frac{H_{f}-H_{o}}{H_{o}}$$

### 2.5. Flexural Test Analysis

**Figure 4:**
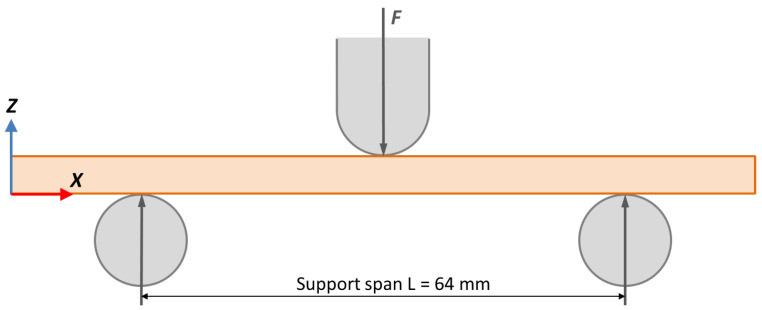
Three-point bending test diagram.

Three-point bending tests (Figure 4) are performed on the heat-treated specimens (with and without mould) to determine the influence of the mould on the mechanical properties.

Tests are performed under ISO 178:2019 standards [35] using an Instrom 5967 (Illinois Tool Works Inc., Glenview, IL, USA) 30 kN load cell with a loading rate of 0.5 mm/s. All specimens are evaluated in the same orientation.

Experimental data are processed to obtain the flexural strain curve graphs and to calculate the maximum flexural stress (flexural strength). The mean and standard deviation of the maximum flexural stress values of the five specimens for each annealing temperature are taken as results (mean ± standard deviation). To obtain the flexural stress in megapascals, Equation (4) is used, where *F* is the load in Newtons, *L* is the span of the support in mm, and *W* and *H* are, respectively, the width and height of each tested specimen in mm.

(4) $$\sigma_{f}=\frac{3\cdot F\cdot L}{2\cdot W\cdot H^{2}}$$

## 3. Results and Discussion

Table 4 and Table 5 show average measurements observed in variations of length (Δ*L*), width (Δ*W*) and height (Δ*H*) for all specimens of each sample. Positive values represent an expansion, whereas negative values represent a shrinkage of every evaluated dimension.

**Figure 5:**
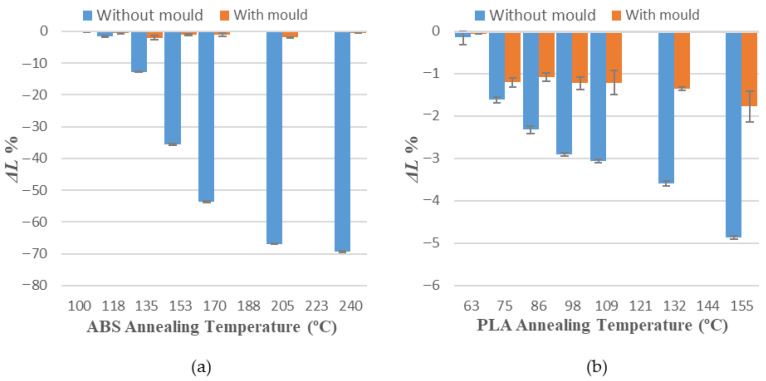
Length variations vs. annealing temperature: (**a**) ABS; (**b**) PLA.

**Figure 6:**
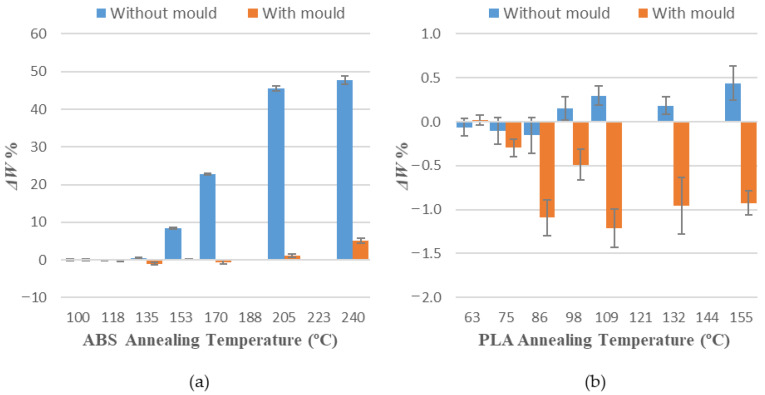
Width variations vs. annealing temperature: (**a**) ABS; (**b**) PLA.

**Figure 7:**
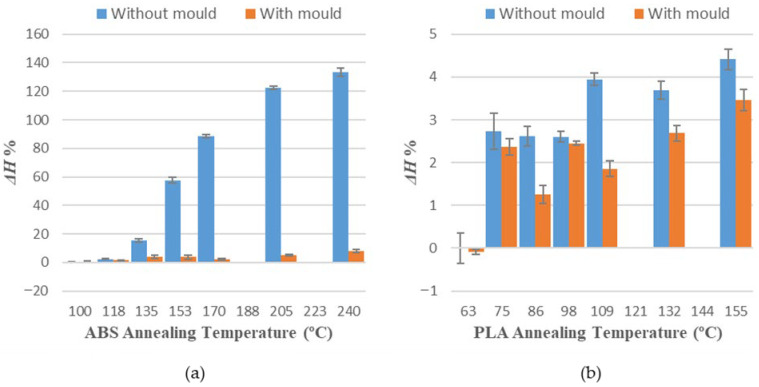
Height variations vs. annealing temperature: (**a**) ABS; (**b**) PLA.

Values obtained for average dimensional variations are represented in a bar chart (Figure 5, Figure 6 and Figure 7). Dimensional variations are directly proportional to the annealing temperature in all tests. ABS shows much higher deformations than PLA in all dimensions studied. It can be observed that the use of a ceramic powder mould has a significant effect on reducing dimensional variations in samples after the thermal treatment in both materials. Nevertheless, using a powder mould has a more significant effect on ABS than on PLA, because ABS undergoes greater deformations.

In ABS and PLA, deformations during heat treatment are more important in the main length of the part (Figure 5), because the direction of the construction line coincides with the length dimension [34]. Similar behaviour can be observed at all annealing temperatures studied in this paper.

### 3.1. Mould Effectiveness to Avoid Deformations at Different Annealing Temperatures

Using a ceramic mould during the thermal post-process allows for a decrease in deformations in both materials (Figure 5, Figure 6 and Figure 7), although length is the most affected dimension for deformation. For this reason, length will be used to evaluate mould effectiveness in both materials.

Mould effectiveness is measured as it is shown in Equation (5), with E being the mould effectiveness to avoid deformations in the length *L* during the thermal post process.

(5) $$E\left(\%\right)=\left(1-\frac{\Delta L_{with\;mould}}{\Delta L_{without\;mould}}\right)\times 100\;$$

A mould effectiveness value (*E*) of 100% means that samples treated with the mould avoid all deformations during the annealing post-process. A value of 0% means that the samples suffer identical deformations with or without the mould.

The mould’s effectiveness for each annealing temperature is shown in Table 6 and Table 7.

**Figure 8:**
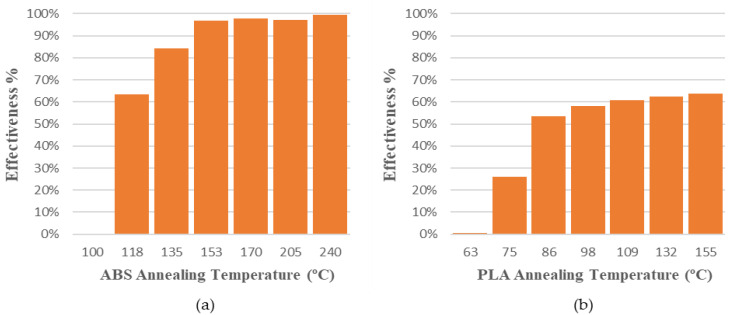
Mould effectiveness vs. annealing temperature to avoid deformations in length dimension: (**a**) ABS; (**b**) PLA.

The values obtained for the mould’s effectiveness are represented in a bar chart (Figure 8). In both cases, the effectiveness of the mould increases with the annealing temperature. Nevertheless, it can also be observed that the mould’s effectiveness is higher in amorphous polymers, or ABS, than in semi-crystalline polymers, or PLA. The annealing temperatures chosen are between the glass transition temperature, Tg, and the melting temperature of the polymers. It is well known that exceeding Tg increases molecular mobility, causing significant changes in thermal properties. Above the glass transition temperature, the polymer tends to expand isotropically, and hysteresis is observed in the expansion or dimensional changes upon cooling [42].

**Figure 9:**
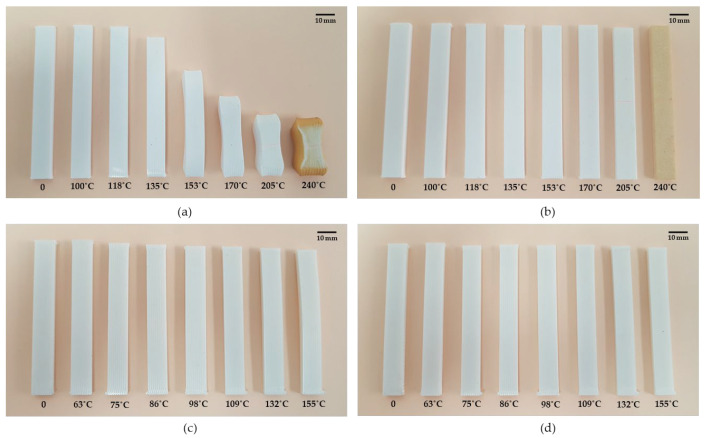
Sample comparison before thermal process (0) and after thermal process at seven annealing temperatures with mould and without mould: (**a**) ABS without mould samples; (**b**) ABS with mould samples; (**c**) PLA without mould samples; (**d**) PLA with mould samples.

Figure 9 shows specimens treated at annealing temperatures studied with and without the mould. ABS presents high deformations and signs of degradation at 240 °C thermal treatment temperature. PLA presents less deformation in all temperatures, and no degradation is observed. The mould’s effectiveness is adequate to reduce deformations in both materials, but the mould is less effective at low annealing temperatures in PLA parts. On the other hand, the mould is highly recommended for ABS parts or parts with high annealing temperatures.

### 3.2. Prediction of Deformations and Mould Effectiviness

**Figure 10:**
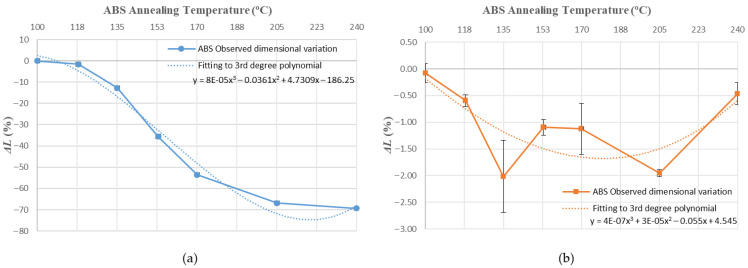
ABS Length variation vs. annealing temperature third-degree polynomial approximation: (**a**) without mould; (**b**) with mould.

**Figure 11:**
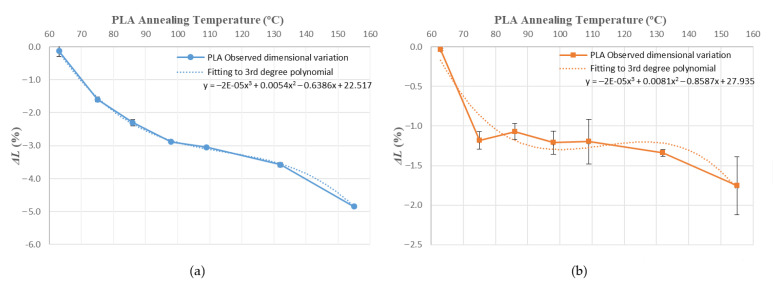
PLA length variation vs. annealing temperature third-degree polynomial approximation: (**a**) without mould; (**b**) with mould.

**Figure 12:**
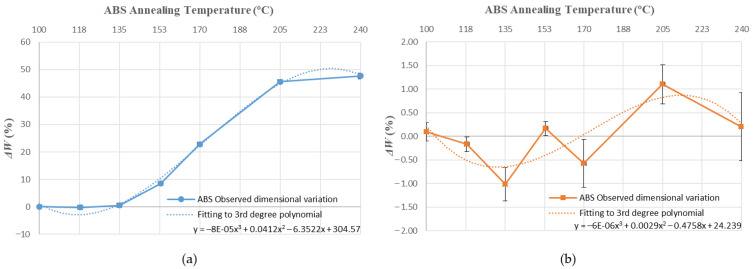
ABS width variation vs. annealing temperature third-degree polynomial approximation: (**a**) without mould; (**b**) with mould.

**Figure 13:**
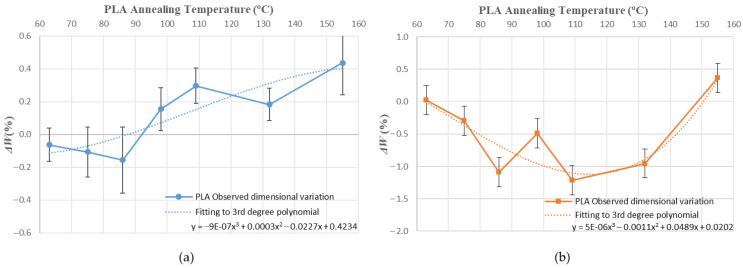
PLA width variation vs. annealing temperature third-degree polynomial approximation: (**a**) without mould; (**b**) with mould.

**Figure 14:**
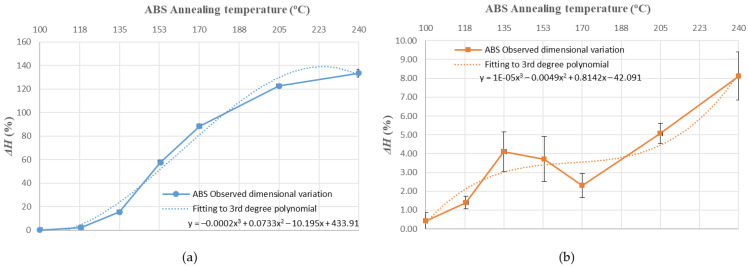
ABS height variation vs. annealing temperature third-degree polynomial approximation: (**a**) without mould; (**b**) with mould.

**Figure 15:**
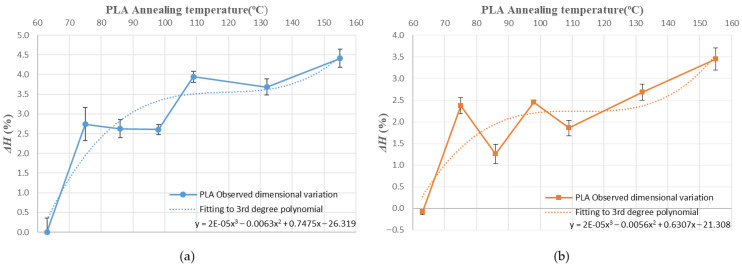
PLA height variation vs. annealing temperature third-degree polynomial approximation: (**a**) without mould; (**b**) with mould.

**Figure 16:**
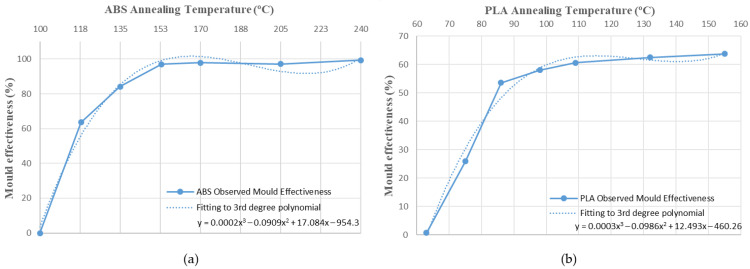
Mould effectiveness vs. annealing temperature third-degree polynomial approximation: (**a**) ABS; (**b**) PLA.

Figure 10, Figure 11, Figure 12, Figure 13, Figure 14, Figure 15 and Figure 16 approximate the results to a polynomial function to predict dimensional deformations and mould effectiveness of ABS and PLA parts during the annealing post-process. The best results are obtained by fitting a third-degree polynomial function.

The polynomial approximation is less precise when a mould is used, because deformations are minor in samples with the mould, as measurement error is greater. Nonetheless, the adjustment to a polynomial of third degree is still correct.

The third degree polynomial fitting of each graph can be expressed in equation form to obtain variations of each dimension as a function of the annealing temperature (Table 8 and Table 9).

As shown in Figure 16 the mould’s effectiveness also fits well to the third polynomial in both materials studied. Therefore, it can be expressed in equation form (Table 10).

The behaviour of the mould at different temperatures is similar in both materials, despite the difference between amorphous (ABS) and semi-crystalline (PLA) materials (Table 10). They differ in the scale on the effectiveness axis, which is smaller in semi-crystalline materials such as PLA. It can be concluded that dimensional variation and mould effectiveness are predictable at different annealing temperatures for ABS and PLA.

### 3.3. Mould Influence on the Mechanical Properties at Different Annealing Temperatures

The annealing post-process is performed to improve the mechanical properties of FDM parts. The use of the mould during annealing is highly recommended to avoid deformations, but it could lead to variations in the mechanical properties of the material. For this purpose, the flexural strength of both materials studied at the different annealing temperatures will be analyzed.

**Figure 17:**
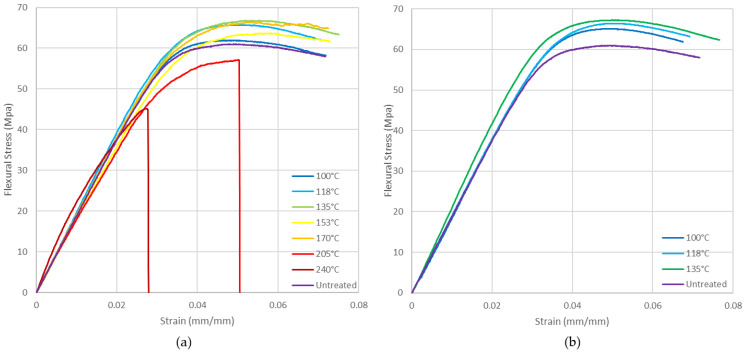
ABS flexural stress vs. strain at different annealing temperatures: (**a**) with mould; (**b**) without mould.

ABS presents a slight improvement in the flexural mechanical properties of the heat-treated specimens compared to the untreated ones (Figure 17). In the treated specimens, improvement is lost from 170 °C and worsens drastically, with premature failure of the specimens occurring from 205 °C onwards. If a mould is not used above 135 °C, the specimen deformations are so high that the flexural test cannot be carried out.

**Figure 18:**
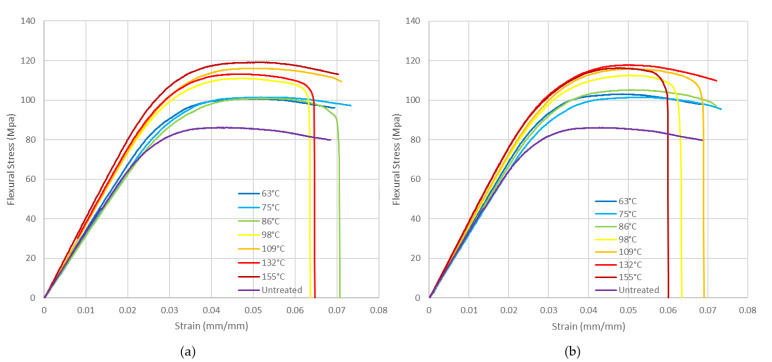
PLA flexural stress vs. strain at different annealing temperatures: (**a**) with mould; (**b**) without mould.

Figure 18 shows PLA flexural stress curves. A significant improvement in flexural mechanical properties is observed at 86 °C and above. Results are similar if mould is not used.

Table 11 and Table 12 show the average maximum flexural stress (flexural strength) of specimens from each sample. In tests 4–7 (Table 11), specimen deformations are so high that the flexural test cannot be carried out.

**Figure 19:**
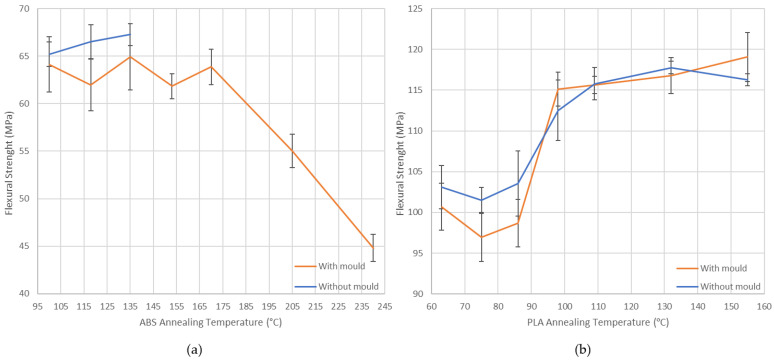
Flexural strength vs. annealing temperatures: (**a**) ABS; (**b**) PLA.

Values obtained for flexural strength are plotted in a graph (Figure 19). Low annealing temperatures using a ceramic powder mould slightly reduce the flexural strength in both materials. In contrast, there is no influence on this mechanical property at high annealing temperatures.

In ABS, at low annealing temperatures, the slight decrease in flexural strength is compensated for by the clear advantage of using a mould to reduce deformations significantly. In PLA, annealing temperatures above 86 °C improve the flexural properties significantly. There is no significant difference in flexural strength if a mould is used or not at these temperatures. On the other hand, using a mould is necessary to reduce part deformations at these temperatures.

An analysis of variance (ANOVA) is performed to quantify the influence of a mould and temperature during post-processing thermal treatment on the flexural strength of 3D-printed samples. An F test with a level significance of 0.05 has been used. Due to strong ABS deformations, if a mould is not used, analysis can only be applied at a temperature range between 100 and 135 °C (Table 13). PLA analysis has been performed for a low temperature range (Table 14) to compare with ABS and to the whole temperature range studied (Table 15).

As “*p* values” are higher than 0.05 in all ANOVAs, there is no significant effect of the factor on the response. For this reason, in all annealing temperature ranges, a mould has no significant influence on the flexural strength of the treated parts, and it can be recommended in both materials.

## 4. Conclusions

In this research, a ceramic powder mould’s influence on the annealing post-process of the two most widely used thermoplastic materials in this type of additive technique—ABS and PLA—has been studied. The annealing post-process has a significant influence on the mechanical properties of FDM-printed parts. However, in this post-process some deformations can appear that can cause the part to be rejected. The present study showed that a ceramic power mould could be a solution to preventing part deformation during the annealing post-process.

Length, width and height specimen variation during the annealing process at different temperatures and mould usage effectiveness have been evaluated. Deformations are directly proportional to the annealing temperature in all tests. ABS shows much higher deformations than PLA in all dimensions studied. However, length is the dimension most affected by deformation; therefore, the effectiveness of the mould has been evaluated mainly according to the length of the part. Usage of the ceramic powder mould has a great effect in reducing deformations in both materials. The mould’s effectiveness increases with annealing temperature. Its behaviour at different temperatures is similar in both materials, although the mould’s effectiveness is higher in amorphous polymers (ABS) than in semi-crystalline polymers (PLA). Dimensional variations and mould effectiveness have been fit to third order polynomial equations in order to predict them as a function of the annealing temperature and material used. 

The flexural strength of ABS and PLA was tested. A wide range of annealing treatment temperatures and ceramic powder mould usage were considered. An analysis of variance (ANOVA) was performed to study the mould’s influence on flexural strength during the annealing treatment. It was found that using this kind of mould during thermal post-processing had no significant impact on flexural strength. It can be concluded that in order to avoid deformations in the annealing of ABS and PLA thermoplastics parts, the usage of a powder mould during annealing is highly recommended.

## Figures and Tables

**Table 1:** Printing parameters.

Material	Printing Speed (mm/s)	Printing Temperature (°C)	Build Plate Temperature (°C)
ABS	60	240	80
PLA	60	215	60

**Table 2:** Variables and levels.

Level	1	2	3	4	5	6	7
Annealing temperature (°C)	100 (63)	118 (75)	135 (86)	153 (98)	170 (109)	205 (132)	240 (155)
Ceramic mould	NO	YES					
Material	ABS	PLA					

**Table 3:** ABS and PLA experiment conditions.

Material	T_annealing_ (°C)	Ceramic Mould	Code	Material	T_annealing_ (°C)	Ceramic Mould	Code
ABS	100	No	1	PLA	63	No	1
		Yes	8			Yes	8
	118	No	2		75	No	2
		Yes	9			Yes	9
	135	No	3		86	No	3
		Yes	10			Yes	10
	153	No	4		98	No	4
		Yes	11			Yes	11
	170	No	5		109	No	5
		Yes	12			Yes	12
	205	No	6		132	No	6
		Yes	13			Yes	13
	240	No	7		155	No	7
		Yes	14			Yes	14

**Table 4:** ABS average dimensional changes after thermal treatment.

Annealing Temperature		Set 1 (Without Mould)				Set 2 (With Mould)		
	Test	Δ*L* (%)	Δ*W* (%)	Δ*H* (%)	Test	Δ*L* (%)	Δ*W* (%)	Δ*H* (%)
100	1	−0.02	0.00	0.34	8	−0.08	0.09	0.41
118	2	−1.63	−0.21	2.30	9	−0.59	−0.17	1.40
135	3	−12.76	0.53	15.53	10	−2.02	−1.01	4.10
153	4	−35.60	8.43	57.68	11	−1.10	0.16	3.71
170	5	−53.55	22.78	88.40	12	−1.13	−0.57	2.31
205	6	−66.85	45.59	122.58	13	−1.95	1.10	5.07
240	7	−69.36	47.72	133.40	14	−0.46	5.13	8.12

**Table 5:** PLA average dimensional changes after thermal treatment.

Annealing Temperature		Set 1 (Without Mould)				Set 2 (With Mould)		
	Test	Δ*L* (%)	Δ*W* (%)	Δ*H* (%)	Test	Δ*L* (%)	Δ*W* (%)	Δ*H* (%)
63	1	−0.13	−0.06	0.00	8	−0.03	0.02	−0.08
75	2	−1.60	−0.11	2.74	9	−1.18	−0.30	2.37
86	3	−2.30	−0.16	2.62	10	−1.07	−1.09	1.26
98	4	−2.88	0.15	2.60	11	−1.21	−0.49	2.45
109	5	−3.05	0.30	3.94	12	−1.20	−1.21	1.86
132	6	−3.58	0.18	3.69	13	−1.34	−0.95	2.69
155	7	−4.84	0.44	4.41	14	−1.76	−0.93	3.46

**Table 6:** ABS mould effectiveness.

Annealing Temperature (°C)	Mould Effectiveness E (%)
100	0.00
118	63.54
135	84.20
153	96.92
170	97.90
205	97.08
240	99.33

**Table 7:** PLA mould effectiveness.

Annealing Temperature (°C)	Mould Effectiveness E (%)
63	0.74
75	25.97
86	53.61
98	58.05
109	60.67
132	62.54
240	63.76

**Table 8:** ABS deformation as a function of annealing temperature.

without Mould	with Mould
Δ*L* (%) = 8 × 10^−5^T^3^ − 0.0361T^2^ + 4.7309T − 186.25	Δ*L* (%) = 4 × 10^−7^T^3^ + 3 × 10^−5^T^2^ − 0.055T + 4.545
Δ*W* (%) = −8 × 10^−5^T^3^ + 0.0412T^2^ − 6.3522T + 304.57	Δ*W* (%) = −6 × 10^−6^T^3^ + 0.0029T^2^ − 0.4758T + 24.239
Δ*H* (%) = −0.0002T^3^ + 0.0733T^2^ − 10.195T + 433.91	Δ*H* (%) = 1 × 10^−5^T^3^ − 0.0049T^2^ + 0.8142T − 42.091

**Table 9:** PLA deformation as a function of annealing temperature.

without Mould	with Mould
Δ*L* (%) = −2 × 10^−5^T^3^ + 0.0054T^2^ − 0.6386T + 22.517	Δ*L* (%) = −2 × 10^−5^T^3^ + 0.0081T^2^ – 0.8587T+ 27.935
Δ*W* (%) = −9 × 10^−7^T^3^ + 0.0003T^2^ − 0.0227T + 0.4234	Δ*W* (%) = 5 × 10^−6^T^3^− 0.0011T^2^ + 0.0489T + 0.0202
Δ*H* (%) = 2 × 10^−5^T^3^ − 0.0063T^2^ + 0.7475T − 26.319	Δ*H* (%) = 2 × 10^−5^T^3^ – 0.0056T^2^ + 0.6307T – 21.308

**Table 10:** Mould effectiveness as a function of annealing temperature.

ABS	PLA
E (%) = 0.0002T^3^ − 0.0909T^2^ + 17.084T − 954.33	E (%) = 0.0003T^3^ − 0.0986T^2^ + 12.493T − 460.26

**Table 11:** ABS flexural strength.

Annealing Temperature	Set 1 (Without Mould)		Set 2 (With Mould)	
	Test	Flexural Strength (MPa)	Test	Flexural Strength (MPa)
100	1	65.1	8	64.1
118	2	66.5	9	61.9
135	3	67.2	10	64.9
153	4	-	11	61.8
170	5	-	12	63.8
205	6	-	13	55.0
240	7	-	14	44.8

**Table 12:** PLA flexural strength.

Annealing Temperature	Set 1 (Without Mould)		Set 2 (With Mould)	
	Test	Flexural STRENGTH (MPa)	Test	Flexural Strength (MPa)
63	1	103.0	8	100.6
75	2	101.4	9	96.9
86	3	103.5	10	98.6
98	4	112.5	11	115.1
109	5	115.7	12	115.6
132	6	117.7	13	116.7
155	7	116.2	14	119.0

**Table 13:** ANOVA for ABS flexural strength (annealing temperature range 100–135 °C).

Source of Variation	Sum of Squares	Df	Mean Square	F Ratio	*p* Value
Mould RESIDUALS	4.68882 177.366	1 22	4.68882 8.06207	0.58	0.4538
TOTAL (CORRECTED)	182.054	23			

**Table 14:** ANOVA for PLA flexural strength (annealing temperature range 63–86 °C).

Source of Variation	Sum of Squares	Df	Mean Square	F Ratio	*p* Value
Mould	44.7999	1	44.7999	2.63	0.1194
RESIDUALS	375.444	22	17.0656		
TOTAL (CORRECTED)	420.244	23			

**Table 15:** ANOVA for PLA flexural strength (annealing temperature range 63–155 °C).

Source of Variation	Sum of Squares	Df	Mean Square	F Ratio	*p* Value
Mould	1.68718	1	1.68718	0.01	0.9151
RESIDUALS	7932.29	54	146.894		
TOTAL (CORRECTED)	7933.98	55			

## Data Availability

Not applicable.
